# Extent of aging across education and income subgroups in Thailand: Application of a characteristic-based age approach

**DOI:** 10.1371/journal.pone.0243081

**Published:** 2020-12-08

**Authors:** Wiraporn Pothisiri, Orawan Prasitsiriphon, Wichai Aekplakorn

**Affiliations:** 1 College of Population Studies, Chulalongkorn University, Pathumwan, Bangkok, Thailand; 2 Health Insurance System Research Office, Health System Research Institute, Bang Khen, Nonthaburi, Thailand; 3 Department of Community Medicine, Faculty of Medicine, Ramathibodi Hospital, Mahidol University, Ratchathewi, Bangkok, Thailand; National Institute of Health and Nutrition, National Institutes of Biomedical Innovation, Health and Nutrition, JAPAN

## Abstract

**Aim:**

This study aimed to identify differences in physical performance across various socioeconomic groups within an older population and to convert those differences into a common metric to facilitate comparisons of aging speed across socioeconomic subgroups.

**Methods:**

We employed data from the 2009 National Health Examination Survey of Thailand. Physical performance was assessed using three health characteristics: grip strength, as a measure of upper body strength; walking speed, as a measure of lower body strength; and a combined measure of grip strength and walking speed, to capture the strength of the whole body. Education level and income were used to distinguish socioeconomic subpopulations. We followed a characteristic-based age approach to transform these population characteristics, which were measured in different units, into a common and comparable aging metric, referred to as *α* − *age*.

**Results:**

Physical aging trajectories varied by sex and socioeconomic status. Some education, particularly secondary or higher education levels, was significantly associated with greater physical strength in older age for both men and women, whereas higher income was significantly associated with physical strength only for men. Across the three health characteristics, having a primary education slowed age-related declines by up to 6.3 years among men and 2.8 years among women, whereas being in a higher income group slowed age-related declines by 8.2 years among men and up to 4.9 years among women.

**Conclusions:**

This study adds new evidence from a developing Asian country regarding the difference in aging speeds across subpopulations associated with different levels of education and income.

## Introduction

The differential aging of population subgroups has gained increasing research attention. This issue is particularly important for developing nations, in which populations are aging rapidly, and health disparities associated with differences in socioeconomic status tend to be major issues. Previous studies, except a recent study by Sanderson et al. [1], employed single-characteristic markers to examine the differences in aging speed. Grip strength and walking speed are among the most commonly assessed physical health characteristics used during aging and health research, to measure upper and lower body strength, respectively. Existing literature has suggested that grip strength and walking speed both decline with increasing age [2–4]. This decline is associated with a variety of adverse health outcomes, including malnutrition and injuries from falls, which can increase the risks of disability and longer hospitalizations (accompanied by higher costs) and, thus, result in reduced health-related quality of life [5–9]. Due to their predictive abilities for all-cause and cause-specific mortality [10–12] and their ease of administration, grip strength and walking speed tests have been widely recommended for inclusion in clinical routines and aging surveys [10, 13, 14]. Although variations in the extent of aging across socioeconomic subgroups have been investigated in various Western populations [15–18], few such studies have been conducted in Asia, especially in developing countries.

This study aimed to investigate differences in aging among different subpopulations in Thailand. The high degree and rapid pace of population aging, combined with the recent worsening socioeconomic inequalities, particularly in terms of income, education, and healthcare [19–21], contribute to making Thailand a particularly compelling location for this study. In addition, Thailand is one of the few Asian countries that maintains national-level survey datasets, including a wide variety of health variables, permitting researchers to use more than one characteristic to measure aging. Based on nationally representative data from the National Health Examination Survey of Thailand, this study identified the physical ages of older subpopulations based on three health characteristics, grip strength, walking speed, and overall body strength, to assess the musculoskeletal strength of both the upper and lower body. To facilitate comparisons, differences in health characteristics were then converted into a common metric (years of age). Previous studies have shown that cohorts of older Thais are socioeconomically heterogeneous [22, 23]; therefore, we expect to observe significant variations in aging according to education and income levels.

## Materials and methods

### Data

The current study collected data from the fourth wave of the cross-sectional National Health Examination Survey of Thailand (NHES-4), which was administered by the Health Systems Research Institute, Ministry of Public Health. The NHES was first conducted in 1991 and has been repeated approximately every 5–6 years since inception. The NHES-4 sample is nationally representative and consists of 31,680 individuals aged 1 year and older. A stratified, multistage sampling approach was used, which involved the initial selection of 21 provinces (including Bangkok), followed by 104 districts, and then 272 villages in rural areas and 340 electoral units in urban areas. Ethical clearance was granted by the Ethical Review Committee for Research in Human Subjects, Thailand Ministry of Public Health. More detailed descriptions of the NHES are available in previous studies [24, 25].

### Study sample

Our study was restricted to individuals aged between 60 and 89 years. Those aged 90 years and older were excluded from this analysis because these individuals tend to represent healthy survivors, and their inclusion could result in selection bias and over-optimistic results. The sample was further restricted to those older individuals who were successfully able to participate in both walking speed and grip strength tests and provided valid information for all of the covariates employed in the analyses. After all restrictions were applied, the final sample comprised 7,847 older adults, accounting for 85.5% of the original sample aged 60–89 years.

### Measurement

*Grip strength* was measured in kilograms using a handgrip dynamometer (Grip-D T.K.K.5401). Prior to the test, the respondents were asked if they had experienced any surgery, injury, or other medical conditions that affected either hand within the previous three months. Only those individuals who did not report any recent history of hand surgery or injury were asked if they were willing to perform the test. During the test, the participants were instructed to sit with elbow flexion at 90 degrees and the forearm and wrist in neutral positions. Then, they were instructed to squeeze the dynamometer as firmly as possible for 3 seconds. Four tests were performed in a row with both hands (2 left and 2 right). The mean grip strength from these 4 trials was recorded and used in this analysis.

*Walking speed* was measured by having participants walk along a 4-meter track at their fastest pace. Respondents aged 60 years and over, with and without assistive devices, were asked if they were willing to participate in this gait test. During the analysis, the reported gait time was converted into speed by dividing four meters by the gait time, in seconds.

*Overall body strength* was a composite measure derived from an average standardized score of grip strength and walking speed. Because these two indices were originally measured using different metrics, we first converted each measure into a Z-score (separately by gender). Then, the two Z-scores were averaged to obtain a single value for the overall index.

### Socioeconomic status

Socioeconomic status was assessed using two variables: education level and income. Education level was incorporated as a categorical variable, indicating whether the respondent had no education, less than primary education, primary education, or secondary or higher education. Reported monthly income was converted into income terciles (high, middle, and low income). No education and the low-income tercile were used as the reference categories during multivariate analyses.

### Other covariates

Other covariates in the analyses included age, anthropometric measures, and physical health status. Age was calculated using the difference between the recorded date of the interview and the date of birth shown on each individual’s identity card or home registration booklet. Age was incorporated as a quadratic continuous variable to account for the nonlinear effects of age on the three physical health characteristics [18, 26]. The inclusion of age squared, instead of age, was verified by three goodness-of-fit measures, including R-squared, the Akaike Information Criterion (AIC), and Bayesian Information Criterion (BIC). The two included anthropometric measures were height and weight, measured in centimeters and kilograms, respectively, which were incorporated as continuous variables.

Physical health status was measured by the presence of multiple chronic diseases, which included cardiovascular disease (CVD), chronic obstructive pulmonary disease (COPD), diabetes and osteoarthritis. The selection of chronic conditions was largely determined by data availability and any significant associations with grip strength or walking speed, as reported by previous studies [10, 27–29]. A dichotomous variable was constructed for each variable based on the self-reporting of medical conditions that have previously been diagnosed by a medical doctor or other health professional. The presence of diabetes was determined based on the self-report of a diabetes diagnosis by a health professional, combined with blood test results and self-reported information regarding the use of glucose-lowering medication within the past 12 months. Respondents with fasting plasma glucose (FPG) values greater than or equal to 126 mg/dl, who reported having been diagnosed with diabetes by a health professional, or who were prescribed glucose-lowering medications by a health professional within the past year were considered to have diabetes.

### Statistical analyses

All analyses were performed using STATA version 12. Sampling weights were applied to account for the multistage sampling design, yielding nationally representative estimates. Descriptive analyses were employed to examine the patterns of the three physical health characteristics, socioeconomic status, and other covariates, according to gender and age group. Then, multivariate analyses were performed to investigate differences in each characteristic across education levels and income terciles, separately by gender. The specification of the models for education took the following form:

$$\begin{array}{l} H{\left(k\right)}_{i}=\beta_{0}+\beta_{1}age^{2}+\beta_{2}Height+\beta_{3}Weight+\beta_{4}Lowprimary+\beta_{5}Primary+\\ \beta_{6}Secondary+\beta_{7}CVD+\beta_{8}COPD+\beta_{9}Diabetes+\beta_{10}Osteoarthritis+\epsilon_{i} \end{array}$$ (1)

and the model for income was as follows:

$$\begin{array}{l} H{\left(k\right)}_{i}=\beta_{0}+\beta_{1}age^{2}+\beta_{2}Height+\beta_{3}Weight+\beta_{4}Middleincome+\beta_{5}Highincome+\\ \beta_{6}CVD+\beta_{7}COPD+\beta_{8}Diabetes+\beta_{9}Osteoarthritis+\epsilon_{i} \end{array}$$ (2)

where *H*(*k*)_*i*_ denotes a health characteristic *k*, referring to the grip strength, walking speed, or overall body strength, of person *i*. *ϵ*_*i*_ represents random error with zero mean and constant variance σ^2^. Prior to performing regression analyses, a Variance Inflation Factor (VIF) test was performed to diagnose the multi-collinearity problem. None of the examined covariates showed VIF values greater than 4, suggesting that the multi-collinearity effect is negligible [30].

To estimate age differences in the physical health characteristics across education levels and income terciles, we followed the characteristic-based age approach described by Sanderson et al. [16], in which we calculated *α* − *ages* for each education level and income tercile. This resulted in the calculation of the predicted mean value of each physical health characteristic, *k*, expressed as a function of chronological age and a set of covariates [16, 18]. For the educational subgroups, *α* − *ages* were computed using the following equations:

$$\alpha_{\text{k},\text{lowprimary}}=\sqrt{{\text{age}}_{\text{noedu}}^{2}-\frac{{\hat{\beta }}_{4}}{{\hat{\beta }}_{1}}}$$ (3)

$$\alpha_{\text{k},\text{primary}}=\sqrt{{\text{age}}_{\text{noedu}}^{2}-\frac{{\hat{\beta }}_{5}}{{\hat{\beta }}_{1}}}$$ (4)

$$\alpha_{\text{k},\text{secondary}}=\sqrt{{\text{age}}_{\text{noedu}}^{2}-\frac{{\hat{\beta }}_{6}}{{\hat{\beta }}_{1}}}$$ (5)

For the income tercile subgroups, *α* − *ages* were computed using the following equations:

$$\alpha_{\text{k},\text{middleinc}}=\sqrt{{\text{age}}_{\text{lowinc}}^{2}-\frac{{\hat{\beta }}_{4}}{{\hat{\beta }}_{1}}}$$ (6)

$$\alpha_{\text{k},\text{highinc}}=\sqrt{{\text{age}}_{\text{lowinc}}^{2}-\frac{{\hat{\beta }}_{5}}{{\hat{\beta }}_{1}}}$$ (7)

For each health characteristic, the *α* − *ages* of older individuals with higher education levels or in higher-income terciles were compared against their less-educated or lower-income counterparts, respectively, who had the same mean values for grip strength, walking speed, or overall body strength. To assess the robustness of the results, we performed sensitivity tests by including cases with missing values that had previously been removed from the primary analyses using imputed values. Assuming that the data were randomly missing, we adopted multiple imputation methods using chain equations to generate 15 imputed datasets. For each health characteristic, missing data were replaced by the mean values or mean scores of the first quintile (1%-20%). The missing data for covariates were imputed using a regression-based method. Table 1 provides an overview of the missing cases for each variable.

**Table 1:** Count and percentage of missing cases, and descriptive statistics of all variables used in the analysis by gender and variable.

Variable	Number of missing cases (%)		Mean (95% CI)/%	
	Men	Women	Men	Women
**Health characteristics**				
Grip strength	64 (1.4%)	66 (1.4%)	26.3 (25.9–26.8)	17.7 (17.4–18.0)
Walking speed	117 (2.6%)	158 (3.4%)	1.1 (1.1–1.2)	1.0 (0.9–1.0)
Overall body strength	157 (3.5%)	206 (4.4%)	0.0 (−1.4–1.4)	0.0 (−1.7–1.7)
**Socioeconomic status**				
Education level	40 (0.9%)	22 (0.5%)		
No education			6.0	16.8
Less than primary			8.1	10.3
Primary			72.3	67.5
Secondary or higher			13.5	5.4
Income tercile	458 (10.2%)	473 (10.1%)		
Low			46.1	43.2
Middle			30.6	28.7
High			23.3	28.1
Age (years)	0 (0.0%)	0 (0.0%)	69.2 (69.0–69.4)	69.1 (68.9–69.4)
Height (cm)	41 (0.9%)	85 (1.8%)	161.2 (160.8–161.6)	150.3 (150.1–150.6)
Weight (kg)	30 (0.7%)	27 (0.6%)	58.2 (57.2–59.2)	53.2 (52.4–54.1)
**Health status (proportion with a chronic condition)**				
CVD	24 (0.5%)	15 (0.3%)	3.8	3.7
COPD	10 (0.2%)	2 (0.0%)	3.3	0.9
Diabetes	168 (3.7%)	156 (3.3%)	13.5	17.1
Osteoarthritis	18 (0.4%)	11 (2.3%)	17.6	28.7
Unweighted N	4,490	4,682	3,701	3,832

Source: Thailand NHES-4.

Note: 95% CI = 95% confidence interval; CVD = Cardiovascular disease; COPD = Chronic obstructive pulmonary disease.

For cases missing information on grip strength and walking speed, our preliminary analysis indicated that the majority of older men and women who chose not to participate in grip and walking tests held primary educations. Older men and women in the low-income tercile constituted the highest proportions of those missing information for the grip and walking tests. However, approximately one-fifth of the older women who chose not to participate in both tests belonged to the high-income tercile, and this proportion was two-fold that of older men in the same tercile (See S1 Table).

## Results

### Overview of the sample

Descriptive statistics for all variables, according to gender, are presented in Table 1. The universe of analysis was defined as the final analytic sample, 49% of whom were older men, and 51% of whom were older women. The average age of both men and women in the sample was 70 years. The average height and weight were 161.2 cm and 58.2 kg for men, respectively, and 150.3 cm and 53.2 kg for women. Most older men and women had at least a primary education. The proportion of older women with no education was 3 times that of men. Across the sample, 46% of older men and 43% of older women were low-income earners. The prevalence of CVD in both older men and women was approximately 3.7%–3.8%. Chronic obstructive pulmonary disease (COPD) was more prevalent among older men than among older women. The proportion of older men diagnosed with COPD was approximately 4-fold that of older women. In contrast, diabetes and osteoarthritis are more common among older women than older men. Approximately 17% and 29% of older women reported being diagnosed with diabetes and osteoarthritis, respectively, compared with 14% and 18% of men. The mean grip strength values were 26.3 kg. for older men and 17.7 kg. for older women. The mean walking speed was similar between older men and women at approximately 1.0–1.1 m/s for both groups.

### Analysis of health characteristics by education

**Fig 1:**
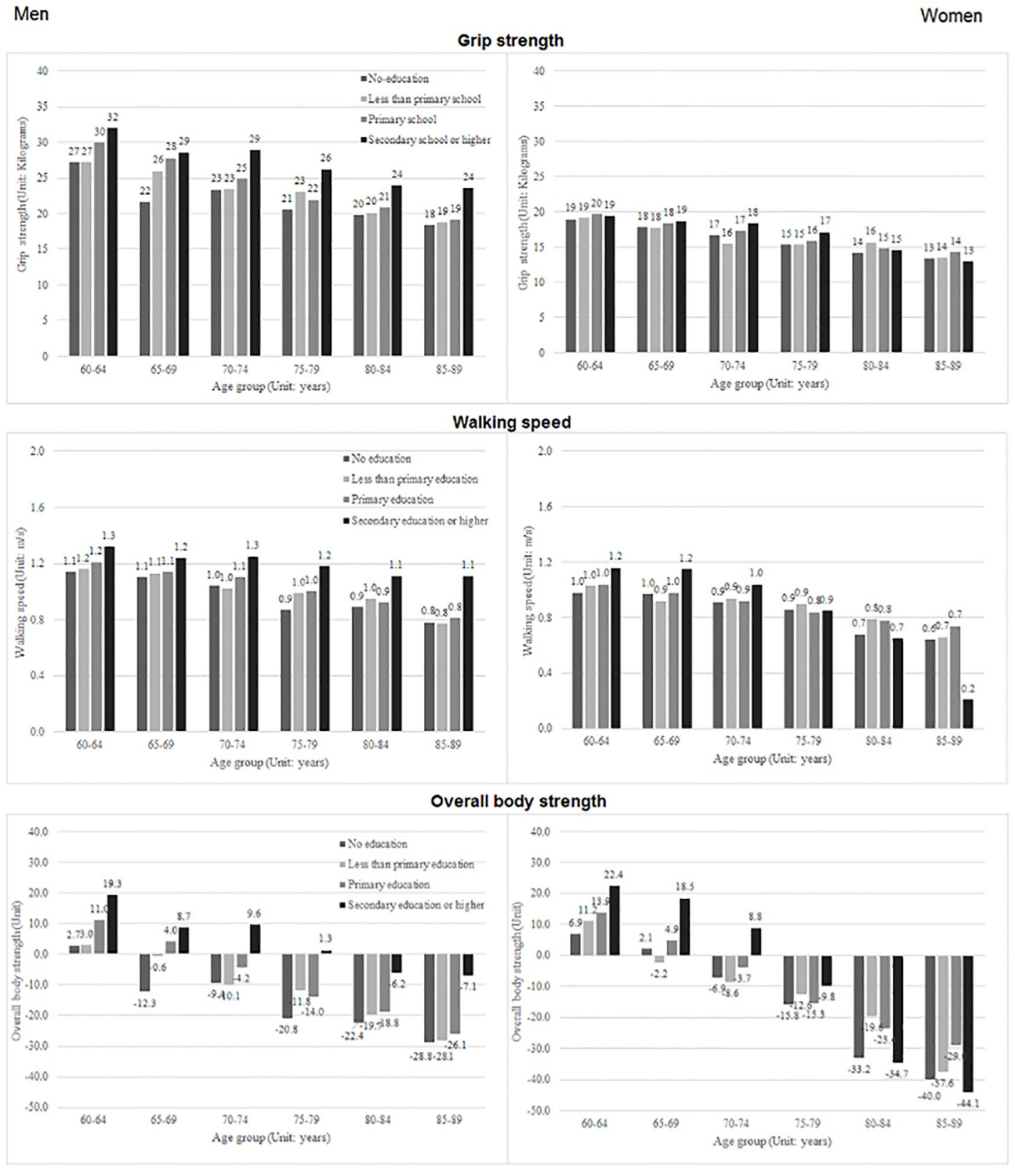
Mean grip strength, walking speed, and overall body strength across education subgroups, according to age and gender.

Fig 1 displays the mean grip strength, walking speed, and overall body strength values for both older men and women, across all age groups and educational levels. The results revealed that for all age groups, older men had stronger grips and faster walking speeds than similarly aged women. The gender difference remained apparent when the two health characteristics were considered together. For older men, the results showed a positive association between education and health characteristics for all age groups. For older women, no clear relationship was observed between grip strength and education level for almost all age groups. A positive association was observed for education and both walking speed and overall body strength among the younger age groups in women. Among the older age groups, particularly the oldest group (85–89 years), the association was reversed for all three health characteristics; older women with at least a secondary education were slightly more likely to have a weaker grip but were considerably more likely to have a slower walking speed and weaker overall body strength than those with lower education levels. This large variation could be due to very few sample size of women in this age group who had attained at least a secondary education.

The positive associations observed between education level and grip strength, walking speed, and overall body strength were broadly supported by the multivariate regression results, which are presented in Table 2. Overall, being better educated, particularly having a secondary education or higher, was associated with better upper, lower, and whole-body strength among both older men (Table 2, Model 1–3) and older women (Table 2, Model 4–6). The education effect was larger for men than for women for all three characteristics. For example, men with secondary education or higher walked, on average, 0.207 m/s faster than their counterparts with no education, whereas women with at least a secondary education walked, on average, 0.145 m/s faster than their counterparts with no education. Table 2 also highlights the significant effects of age squared on grip strength, walking speed, and overall body strength for both men and women, as mentioned previously. The effect of aging on the decline in men’s grip strength was almost twice that observed for women. In contrast, the decline in overall body strength observed as an effect of aging in women was approximately 1.5 times that observed in men. No gender difference was observed for the effects of aging on the decline in walking speed.

**Table 2:** Unstandardized coefficients and standard errors from multivariate regressions addressing education differentials for grip strength (Models 1 and 4), walking speed (Models 2 and 5), and overall body strength (Models 3 and 6).

	Men			Women		
	Model 1	Model 2	Model 3	Model 1	Model 2	Model 3
Age^2^	-0.0024**	-0.0001**	-0.0084**	-0.0012**	-0.0001**	-0.0119**
	0.0001	0.0000	0.0002	0.0001	0.0000	0.0004
Weight	0.1613**	-0.0013**	0.3579**	0.0485**	-0.0019**	0.0305
	0.0128	0.0004	0.0306	0.0068	0.0004	0.0428
Height	0.2215**	0.0009	0.5643**	0.1633**	0.0041**	0.8977**
	0.0155	0.0015	0.0645	0.0098	0.0008	0.0768
Education (Ref.: No education)						
Less than primary	0.9178*	0.0349+	3.1911*	-0.3189	0.0334*	1.4374
	0.4197	0.0196	1.2603	0.2491	0.0140	1.4807
Primary	1.8396**	0.0727**	6.4741**	0.3453+	0.0334**	3.8101**
	0.3050	0.0248	1.2809	0.1662	0.0087	0.8354
Secondary or higher	3.3279**	0.2070**	13.7819**	0.3918+	0.1448**	12.5731**
	0.3403	0.0253	1.2490	0.2104	0.0138	1.3969
CVD (Ref.: without)						
	-1.5387**	-0.0822*	-6.0022**	-1.0765*	-0.0080	-4.4621
	0.3585	0.0313	1.5780	0.3778	0.0237	2.8434
COPD (Ref.: without)						
	-1.0436**	0.0566+	-0.9910	-0.8241*	0.0678*	2.2882
	0.3519	0.0270	1.2501	0.3448	0.0317	2.2978
Diabetes (Ref.: without)						
	-1.9109**	-0.0189	-5.1727**	-1.2335**	-0.0637**	-9.3260**
	0.2603	0.0122	0.7110	0.1443	0.0096	0.9161
Osteoarthritis (Ref.: without)						
	-1.2602**	-0.1209**	-6.3839**	-0.4985*	-0.0449**	-5.2448**
	0.1994	0.0330	1.0886	0.1725	0.0102	1.2229
Constant	-8.3476**	1.4311**	-76.0006**	-3.2080+	0.8948**	-79.2453**
	2.4915	0.2491	10.1420	1.5611	0.1132	11.6756
R-square	40.77%	14.41%	38.32%	23.78%	17.92%	27.34%
Unweighted N	3,701	3,701	3,701	3,832	3,832	3,832

Source: Thailand NHES-4.

Note: CVD = Cardiovascular disease and COPD = Chronic obstructive pulmonary disease;

** p < 0.01,

* p < 0.05,

^+^ p < 0.10.

The conversion of the effects of educational differences into *α* − *ages* is presented in Table 3. For each characteristic, we reported the *α* − *ages* for older men and women according to three education levels: less than primary education, primary education, and secondary education or higher. The patterns observed for men and women with no education were used as the standard for comparison for all characteristics. The results showed that men aged 70.6 years with secondary education or higher, men aged 66.0 years with primary education, and men aged 60 years with no education had the same grip strength. Women aged 62.6 years with secondary education or higher, women aged 62.3 years with primary education, and women aged 60 with no education had the same grip strength. The differences in *α* − *ages* across education subgroups appeared to decline for older age groups. At 85 years, older men with no education had the average grip strength of men with primary education who were 4.4 years older and men with a secondary education or higher who were 7.8 years older. A similar pattern was observed among older women. Women aged 85 years had the same grip strength as women 1.6 years older with primary education and women 1.8 years older with secondary education or higher.

**Table 3:** Alpha-age for men and women by education level.

Age	Men			Women		
	Less than primary	Primary	Secondary or higher	Less than primary	Primary	Secondary or higher
	**Grip strength**					
60	**63.1**	**66.0**	**70.6**	57.8	**62.3**	**62.6**
65	**67.9**	**70.6**	**74.9**	63.0	**67.1**	**67.4**
70	**72.7**	**75.2**	**79.2**	68.2	**71.9**	**72.2**
75	**77.5**	**79.9**	**83.7**	73.3	**76.8**	**77.1**
80	**82.3**	**84.6**	**88.2**	78.4	**81.7**	**81.9**
85	**87.2**	**89.4**	**92.8**	83.5	**86.6**	**86.8**
	**Walking speed**					
60	**63.1**	**66.3**	**76.7**	**62.8**	**62.8**	**71.4**
65	**67.9**	**70.9**	**80.7**	**67.6**	**67.6**	**75.7**
70	**72.7**	**75.5**	**84.7**	**72.4**	**72.4**	**80.0**
75	**77.5**	**80.2**	**88.9**	**77.3**	**77.3**	**84.4**
80	**82.4**	**84.9**	**93.2**	**82.1**	**82.1**	**88.9**
85	**87.2**	**89.6**	**97.5**	**87.0**	**87.0**	**93.4**
	**Overall body strength**					
60	**63.1**	**66.1**	**72.4**	61.0	**62.6**	**68.2**
65	**67.9**	**70.7**	**76.6**	65.9	**67.4**	**72.7**
70	**72.7**	**75.3**	**80.9**	70.9	**72.2**	**77.2**
75	**77.5**	**80.0**	**85.3**	75.8	**77.1**	**81.7**
80	**82.3**	**84.7**	**89.7**	80.8	**82.0**	**86.3**
85	**87.2**	**89.4**	**94.2**	85.7	**86.9**	**91.0**

Note: Alpha-ages shown in bold are significant at p-value = 0.10.

The *α* − *ages* derived from walking speed showed consistent patterns. Men aged 60 years with no education walked at the same speed as those with some primary education who were 3.1 years older, those with primary education who were 6.3 years older, and those with at least a secondary education who were 16.7 years older. The gaps in *α* − *ages* across education groups were narrower at older ages. At age 85, men with no education had the same average walking speed as men with at least some education who were 2.2–12.5 years older. Uneducated women at age 60 walked at the same speed as educated women 2.8–11.4 years older, and the difference in *α* − *ages* was reduced to 2.0–8.4 years for educated women compared with uneducated women at age 85 (Table 3).

The pattern observed for *α* − *ages* based on the combination of two health characteristics was similar to the patterns observed for each characteristic individually. At age 60, the differences in *α* − *age* across education subgroups were 3.1–12.4 years for men and 2.6–8.2 years for women. At age 85, these differences decreased to 2.2–9.2 years for men and 1.9–6.0 years for women.

### Analysis of health characteristics by income

**Fig 2:**
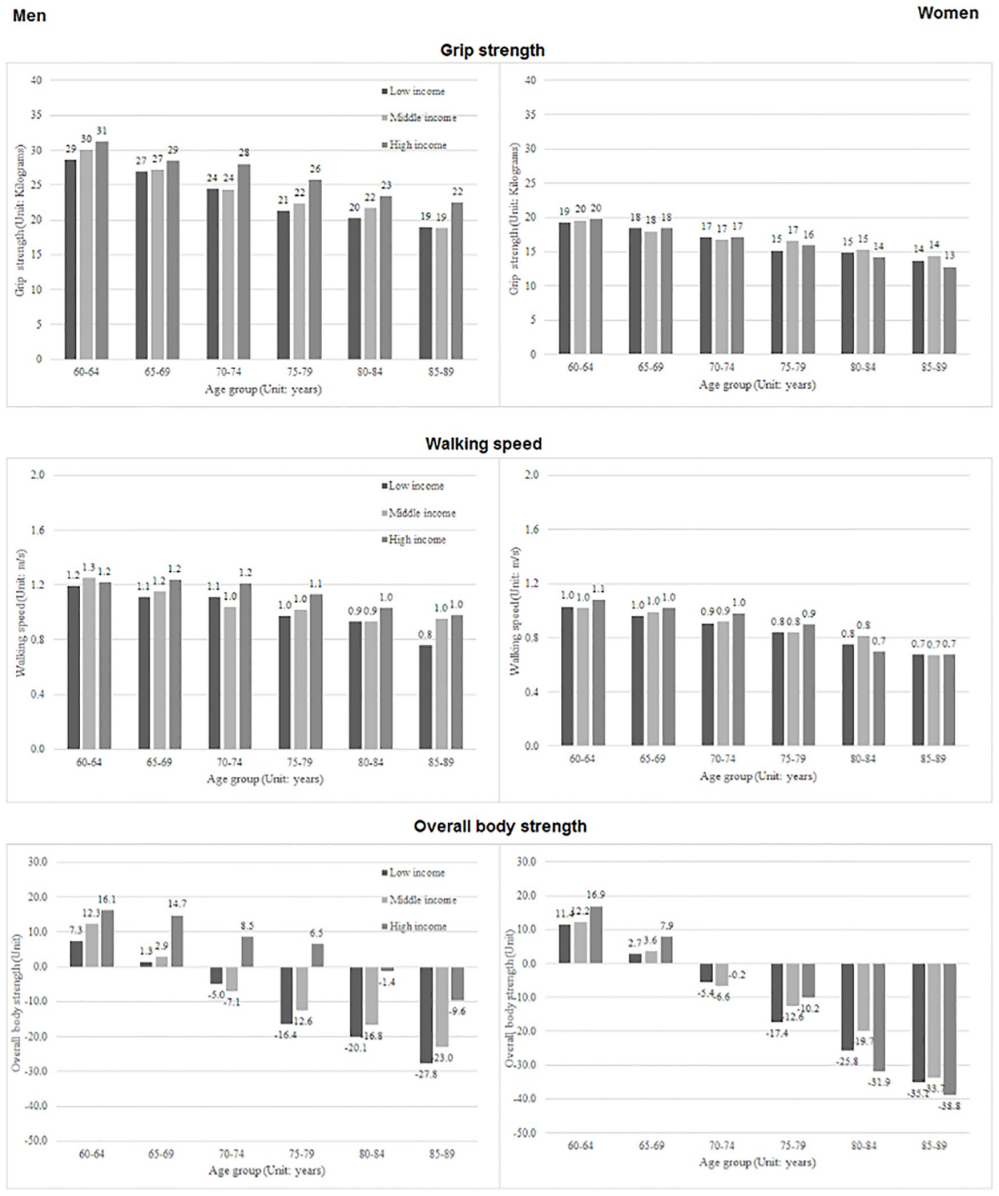
Mean grip strength, walking speed, and overall body strength across income subgroups, according to age and gender.

The mean values of grip strength, walking speed, and overall body strength among older men and women across age and income groups are illustrated in Fig 2. For older men, all characteristics showed a consistent pattern, with positive effects associated with increased income observed for all age groups, whereas for older women, this association was observed only at the younger ages. The absence of positive income effects among the older age groups, particularly those aged 85–89 years, could be due to the relatively small sample size for this older population among the high-income group.

These observations were broadly supported by the regression results shown in Table 4. Older men in higher economic groups, particularly those in the highest income tercile, had significantly greater grip strength, walking speed, and overall body strength than those in the lower economic groups (Table 4, Model 1–3). A similar pattern was observed for walking speed and overall body strength among older women (Table 4, Model 4–6).

**Table 4:** Unstandardized coefficients and standard errors from multivariate regressions addressing income differentials for grip strength (Models 1 and 4), walking speed (Models 2 and 5), and overall body strength (Models 3 and 6).

	Men			Women		
	Model 1	Model 2	Model 3	Model 1	Model 2	Model 3
Age^2^	-0.0024**	-0.0001**	-0.0083**	-0.0013**	-0.0001**	-0.0121**
	0.0001	0.0000	0.0003	0.0001	0.0000	0.0005
Weight	0.1617**	-0.0012*	0.3609**	0.0515**	-0.0020**	0.0315
	0.0123	0.0004	0.0293	0.0067	0.0004	0.0423
Height	0.2251**	0.0012	0.5806**	0.1649**	0.0039**	0.8924**
	0.0158	0.0016	0.0673	0.0095	0.0008	0.0756
Income (Ref.: Low income)						
Middle income	0.4180	0.0249	1.7018	-0.1272	0.0122	0.4874
	0.3242	0.0149	1.1462	0.1552	0.0079	0.8510
High income	1.5129**	0.0941**	6.2641**	-0.2471	0.0584**	3.6209**
	0.1886	0.0116	0.6564	0.1889	0.0116	1.1844
CVD (Ref.: without)						
	-1.3097**	-0.0646*	-4.9618**	-1.0382*	-0.0074	-4.2840
	0.3656	0.0291	1.5249	0.3838	0.0234	2.8505
COPD (Ref.: without)						
	-1.0731**	0.0531+	-1.1591	-0.8125*	0.0606+	1.7714
	0.3679	0.0257	1.2370	0.3350	0.0312	2.2556
Diabetes (Ref.: without)						
	-1.8732**	-0.0152	-4.9805**	-1.2391**	-0.0629**	-9.2789**
	0.2685	0.0133	0.7505	0.1432	0.0100	0.9438
Osteoarthritis (Ref.: without)						
	-1.2854**	-0.1235**	-6.5171**	-0.4810*	-0.0452**	-5.2108**
	0.2048	0.0356	1.2169	0.1692	0.0103	1.2152
Constant	-7.6786**	1.4276**	-74.4692**	-2.9613+	0.9352**	-75.2482**
	2.5553	0.2477	10.5110	1.6438	0.1116	11.8675
R-square	40.31%	13.07%	37.36%	23.58%	17.50%	26.81%
Unweighted N	3,701	3,701	3,701	3,832	3,832	3,832

Source: Thailand NHES-4.

Note: CVD = Cardiovascular disease; COPD = Chronic obstructive pulmonary disease;

** p < 0.01,

* p < 0.05,

^+^ p < 0.10.

The conversion of income differences into years of age is reported in Table 5. The patterns and characteristics observed for men and women in the lowest income tercile were used as the standard for comparisons. The results indicated that low-earning men aged 60 years had the same average grip strength as high-earning men aged 65.1 years, the same walking speed as high-income earners aged 68.2 years, and the same overall body strength as high-income earners aged 66.0 years. Similar to education, the differences in *α* − *ages* for grip strength in men across income subgroups decreased with age. However, a consistent pattern was observed for the other two characteristics. Low-income men at age 85 years had the same grip strength as high-earning men 4.3 years older. The *α* − *ages* for women, based on walking speed, showed an advantage of 4.9 years for high-income earners. Similarly, income differentials at age 85 reduced to 3.5 years. The advantage of having a high income on women’s overall body strength was 2.4 years at age 60 and 1.7 years at age 85 (Table 5).

**Table 5:** Sex-specific alpha-age by income tercile.

Age	Men		Women	
	Middle	High	Middle	High
	**Grip strength**			
60	61.4	**65.1**	59.2	58.4
65	66.3	**69.7**	64.3	63.5
70	71.2	**74.4**	69.3	68.6
75	76.2	**79.1**	74.4	73.7
80	81.1	**83.9**	79.4	78.8
85	86.0	**88.6**	84.4	83.9
	**Walking speed**			
60	62.3	**68.2**	61.0	**64.9**
65	67.1	**72.7**	66.0	**69.5**
70	72.0	**77.2**	70.9	**74.2**
75	76.8	**81.7**	75.8	**78.9**
80	81.7	**86.3**	80.8	**83.7**
85	86.6	**91.0**	85.7	**88.5**
	**Overall body strength**			
60	61.7	**66.0**	60.3	**62.4**
65	66.6	**70.6**	65.3	**67.3**
70	71.5	**75.2**	70.3	**72.1**
75	76.4	**79.9**	75.3	**77.0**
80	81.3	**84.6**	80.3	**81.8**
85	86.2	**89.3**	85.2	**86.7**

Note: Alpha-ages shown in bold are significant at p-value = 0.10.

### Robustness of the results

All multivariate regression analyses were repeated using imputed values for missing data and yielded essentially the same results as those performed using analytic data. Similarly, the *α* − *ages* were recalculated for each health characteristic and socioeconomic subgroup, and the results were similar to the main results (S2 and S3 Tables).

## Discussion

This study is among the first conducted in a developing country that attempts to demonstrate the effects of socioeconomic inequalities on the speed of aging using data from a large-scale, population-based survey. In line with previous studies, our study found that socioeconomic status is significantly and positively correlated with better physical performance, as reflected by three health measures, grip strength, walking speed, and a combined measure of the two. More specifically, our study demonstrated that education level, particularly secondary education or higher, was significantly associated with improved physical strength among older age groups. This observation was true for both men and women and agreed with the reported findings of a number of previous studies [18, 31–34]. When examining the effects of socioeconomic status, our study found that income was significantly associated with all three health characteristics for older men. For older women, in contrast, we do not find evidence of a relationship between income and grip strength. This finding agrees with the findings reported by a prior study based on Survey of Health, Ageing and Retirement in Europe (SHARE) data, which found that income was significantly associated with grip strength among older European men but not women [35].

Translating the effects of education and income differences on physical performance into years of age provided clear evidence supporting variations in the speed of aging among socioeconomic subgroups. Across the three health characteristics, having a primary education appeared to slow the speed of aging by 6.3 years in men and 2.8 years in women. The benefit increases considerably, to 16.7 years in men and 11.4 years in women, among older adults who completed secondary education or higher. Although the number of years gained from education increased with education level, this difference decline with increasing age, to approximately 2.2–12.5 years in men and 2.0–8.1 years in women. When examining the effects of income, the advantage of being in a higher income tercile was moderately lower than the advantage associated with higher education levels. Across the three measured health characteristics, being in a high-income group appeared to delay the effects of aging by 8.2 years in men and up to 4.9 years in women. Similar to education, the magnitude of this difference decreased with advancing age, to 3.6–6.0 years in men and 1.7–3.5 years in women.

This study is not without limitations. First, the cross-sectional nature of the NHES-4 restricts the study from addressing causalities between socioeconomic status (i.e., education and income) and physical health performance. For education, the fact that participants’ education levels were achieved long before the survey suggests the former impacted the latter, but we cannot rule out the potential effects of additional factors that may connect these characteristics. In contrast, the poor physical performance represented by weak grip strength or walking speed may be directly associated with low incomes. Without knowing the baseline income level, our design cannot easily disentangle whether income is a cause or consequence of physical performance in old age. Our study also cannot identify whether a multiple-characteristic indicator is better than a single-characteristic indicator. In a recent study reported by Sanderson et al. [1], survivorship was used as one of the goal characteristics used to examine the advantages of combining grip strength and chair rise speed. Information regarding survivorship was drawn from several waves of the English Longitudinal Survey of Aging (ELSA), between 2004 and 2012. The inability of our cross-sectional study to perform this type of evaluation underscores the need for Thailand to collect panel data so that the mechanisms that link socioeconomic status and health characteristics can be addressed. Furthermore, this survey did not include older persons living in institutional settings (e.g., nursing homes, public and private assisted-living facilities, and jails). However, the impacts of such exclusion appear likely to be minimal, as institutionalized older persons constitute less than 0.01% of the total older population [36]. Some older individuals may have refused to perform the tests due to physical limitations, illness, or busy schedules. Although these missing data were imputed, the potential exists for the overestimation of grip strength and walking speed among this population, which would result in the underestimation of the effects of aging. Finally, we acknowledge that our analyses did not include other health-related variables, such as activities of daily living, functional limitations, and health-related behaviors, that could directly and indirectly affect the physical performance of older persons. Therefore, future studies should consider other potential covariates during the computation of *α* − *ages*.

## Conclusions

Future generations of older adults in Thailand will likely differ from the current generations with respect to education, income, and health status [37]. Based on the findings from our study, these changes will likely result in better physical functioning and, thus, greater advantages in aging speed as measured in years of age. However, sound social policies must be enacted by the government to ensure the improvement of socioeconomic status, nationwide, and across all subpopulations.

## Supporting information

S1 Table Percentage distribution of the older sample who opted not to participate in grip and walking speed tests, according to education level, income, and gender. (PDF)

S2 Table Alpha-age for men and women by education level, using imputed values for missing data. (PDF)

S3 Table Sex-specific alpha-age by income tercile, using imputed values for missing data. (PDF)
