# Supplementary material for: Extent of aging across education and income subgroups in Thailand: Application of a characteristic-based age approach
Source: PLoS One. 2020 Dec 8;15(12):e0243081. doi: 10.1371/journal.pone.0243081 (PMC7723296; doi:10.1371/journal.pone.0243081)
Supplement: S1 Table — (PDF) [file pone.0243081.s001.pdf]

S1 Table. Percentage distribution of older sample who did not participate in grip and walking speed tests by education, income and gender

	**Grip test**		**Waking speed test**	
	**Men**	**Women**	**Men**	**Women**
Total unweighted cases	4,493	4,682	4,493	4,682
% did not undertake the test	1.5	1.3	2.7	3.4
Education				
No education	13.7	19.5	8.4	23.8
Less than primary education	8.9	15.0	9.4	6.9
Primary education	56.8	62.1	67.8	61.9
Secondary or higher education	20.6	3.4	14.4	7.5
Total	100.0	100.0	100.0	100.0
Income tercile				
Low	69.6	43.2	67.0	57.8
Middle	12.2	34.8	20.3	20.0
High	18.2	22.0	12.7	22.2
Total	100.0	100.0	100.0	100.0
