# Supplementary material for: Extent of aging across education and income subgroups in Thailand: Application of a characteristic-based age approach
Source: PLoS One. 2020 Dec 8;15(12):e0243081. doi: 10.1371/journal.pone.0243081 (PMC7723296; doi:10.1371/journal.pone.0243081)
Supplement: S2 Table — (PDF) [file pone.0243081.s002.pdf]

S2 Table. Alpha-age for men and women by education level, using imputed values for missing data

Age	Men			Women		
	Less than primary	Primary	Secondary or higher	Less than primary	Primary	Secondary or higher
**Grip strength**						
60	**63.4**	**66.0**	**70.7**	58.9	**62.9**	**63.4**
65	**68.1**	**70.5**	**75.0**	64.0	**67.6**	**68.2**
70	**72.9**	**75.2**	**79.4**	69.1	**72.5**	**73.0**
75	**77.7**	**79.8**	**83.8**	74.2	**77.3**	**77.8**
80	**82.6**	**84.6**	**88.3**	79.2	**82.2**	**82.6**
85	**87.4**	**89.3**	**92.9**	84.3	**87.0**	**87.5**
**Walking speed**						
60	**64.5**	**66.4**	**73.5**	**62.3**	**61.9**	**69.7**
65	**69.1**	**71.0**	**77.6**	**67.1**	**66.8**	**74.0**
70	**73.9**	**75.6**	**81.8**	**72.0**	**71.7**	**78.5**
75	**78.6**	**80.2**	**86.2**	**76.9**	**76.6**	**82.9**
80	**83.4**	**84.9**	**90.5**	**81.7**	**81.5**	**87.5**
85	**88.2**	**89.6**	**95.0**	**86.6**	**86.4**	**92.1**
**Overall body strength**						
60	**63.4**	**65.9**	**72.2**	60.6	**62.3**	**67.6**
65	**68.2**	**70.5**	**76.4**	65.5	**67.1**	**72.1**
70	**72.9**	**75.1**	**80.7**	70.5	**72.0**	**76.6**
75	**77.7**	**79.8**	**85.1**	75.5	**76.9**	**81.2**
80	**82.6**	**84.5**	**89.5**	80.4	**81.7**	**85.9**
85	**87.4**	**89.3**	**94.0**	85.4	**86.6**	**90.5**

Note: Alpha-ages shown in bold face are statistically significant at p-value=0.10.
