# Supplementary material for: Extent of aging across education and income subgroups in Thailand: Application of a characteristic-based age approach
Source: PLoS One. 2020 Dec 8;15(12):e0243081. doi: 10.1371/journal.pone.0243081 (PMC7723296; doi:10.1371/journal.pone.0243081)
Supplement: S3 Table — (PDF) [file pone.0243081.s003.pdf]

S3 Table. Sex-specific alpha-age by income tercile, using imputed values for missing data

Age	Men		Women	
	**Middle**	**High**	**Middle**	**High**
**Grip strength**				
60	62.0	**65.6**	59.4	59.0
65	66.9	**70.2**	64.5	64.1
70	71.7	**74.8**	69.5	69.1
75	76.6	**79.5**	74.5	74.2
80	81.5	**84.3**	79.6	79.2
85	86.4	**89.0**	84.6	84.3
**Walking speed**				
60	62.9	**67.5**	61.4	**64.5**
65	67.6	**72.0**	66.3	**69.2**
70	72.5	**76.5**	71.2	**73.9**
75	77.3	**81.1**	76.1	**78.6**
80	82.2	**85.7**	81.0	**83.4**
85	87.0	**90.4**	86.0	**88.2**
**Overall body strength**				
60	62.2	**66.5**	60.4	**62.2**
65	67.0	**71.0**	65.3	**67.0**
70	71.9	**75.6**	70.3	**71.9**
75	76.8	**80.3**	75.3	**76.7**
80	81.7	**85.0**	80.3	**81.6**
85	86.6	**89.7**	85.3	**86.5**

Note: Alpha-ages shown in bold face are statistically significant at p-value=0.10.
